# Gastrointestinal Stromal Tumor With Chondrosarcomatous Dedifferentiation Following Imatinib Therapy

**DOI:** 10.7759/cureus.17448

**Published:** 2021-08-25

**Authors:** Nektarios Koufopoulos, Andriani Zacharatou, Sophia Athanasiadou, Periklis Tomos, Panagiota Ekonomopoulou, Theodoros Liakakos, Ioannis G Panayiotides

**Affiliations:** 1 2nd Department of Pathology, National and Kapodistrian University of Athens, “Attikon” University Hospital, Athens, GRC; 2 Department of Pathology, “Vardakeion and Proion” General Hospital of Syros, Hermoupolis, GRC; 3 Department of Thoracic Surgery, National and Kapodistrian University of Athens, “Attikon” University Hospital, Athens, GRC; 4 2nd Department of Internal Medicine - Propaedeutic, National and Kapodistrian University of Athens, “Attikon” University Hospital, Athens, GRC; 5 1st Department of Surgery, “Laikon” General University Hospital/National and Kapodistrian University of Athens, School of Medicine, Athens, GRC

**Keywords:** gastrointestinal stromal tumor, dedifferentiation, stomach, cd117, metastasis, imatinib

## Abstract

Gastrointestinal stromal tumors (GISTs) are the most common mesenchymal neoplasms of the digestive tract, followed by schwannomas, lipomas, leiomyomas, and vascular tumors. They arise more often in the stomach, followed by the small bowel, esophagus, and rectum. Imatinib mesylate, a tyrosine kinase inhibitor with activity against ABL, BCR-ABL, platelet-derived growth factor receptor-alpha (PDGFRA), and c-KIT (CD117), constitutes the cornerstone of treatment for inoperable or metastatic GIST. Cases showing disease progression or resistance to imatinib mesylate may retain their morphology or present unusual morphologic and immunohistochemical characteristics.

We herein describe a case of a 67-year-old patient with a previous history of GIST of the stomach, with local recurrence, who was admitted with a workup of lung nodule on chest computed tomography as part of the routine follow-up. The nodule was resected which showed a malignant tumor composedof epithelioid cells, with an abrupt transition to chondrosarcoma. Epithelioid cells were immunostained for CD117, DOG1, and Vimentin, whereas chondrosarcomatous cells expressed only Vimentin. These findings were consistent with metachronous pulmonary metastasis of the previously diagnosed GIST with chondrosarcomatous dedifferentiation. No KIT or PDGFRA mutation was detected. A review of all accessible pertinent papers disclosed 26 similar cases with unusual morphological and immunohistochemical findings, either post-imatinib treatment or, less commonly, de novo, with heterogeneous differentiation.

Awareness of the histological and immunohistochemical changes in GISTs post imatinib therapy is essential to avoid a severe diagnostic pitfall.

## Introduction

Gastrointestinal stromal tumors (GISTs) are the most common mesenchymal tumors of the digestive tract. They originate from or differentiate into interstitial cells of Cajal, which function as pacemaker cells [1]. They usually arise from the stomach, followed by the small bowel, esophagus, and rectum [2]; less often, GISTs have been reported in the gallbladder and appendix [1]. Moreover, so-called extra-gastrointestinal GISTs (E-GISTs) have been reported in the prostate, mesentery, omentum, retroperitoneum, scrotum, bladder, ovary, pancreas, and vagina [3]. Imatinib mesylate (IM), an oral tyrosine kinase inhibitor (TKI) targeting c-KIT (CD117), platelet-derived growth factor receptor-alpha (PDGFRA), and the fusion protein BCR-ABL [3] constitutes first-line standard treatment for locally advanced or metastatic GISTs. Patients with GIST treated with IM show a remarkable initial benefit followed, in the majority of patients, by disease progression or secondary resistance due to acquired mutations in c-KIT or PDGFRA [4]. Disease progression is manifested as general tumor expansion, resistant nodules, or new metastatic lesions [5, 6]. The majority of tumors during disease progression retain their original morphological features, while in a few cases they may develop heterogeneous differentiation [7].

Post-IM treatment dedifferentiation of a spindle cell GIST, demonstrating an abrupt transition to a high-grade sarcoma with loss of CD117 and CD34 expression in the dedifferentiated component, was first described by Pauwels et al. in 2005 [5]; in 2013, Antonescu et al. described de novo GIST dedifferentiation in TKI naïve tumors [8]. Twenty-six cases of dedifferentiated GIST have so far been described [5-16], displaying a wide morphologic and immunophenotypic spectrum of dedifferentiation.

We herewith describe a case of a gastric GIST with metachronous pulmonary metastasis showing heterologous differentiation after therapy with IM; our case is the first (to our best knowledge) with chondrosarcomatous histology, thus adding another morphologic facet to GIST dedifferentiation.

## Case presentation

**Figure 1:**
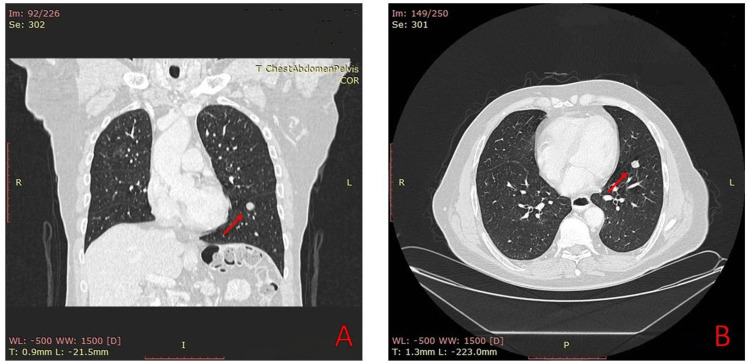
Chest CT revealed a 1 cm lesion in the lower lobe of the left lung (red arrows).

A 67-year-old male was admitted to the Department of Thoracic Surgery in July 2019. Past medical history was significant for a previously (2005) operated, 10 cm large gastric GIST with metastasis to two regional lymph nodes. The patient received adjuvant therapy with IM for 24 months. Surgical resection of a recurrent tumor was performed in 2009, with a further 12-month adjuvant treatment with IM. His clinical course was uneventful until 2018 when a small lesion adjacent to the tail of the pancreas was found on abdominal computed tomography (CT). This lesion was stable until 2019 when a 1 cm tumor was found in the left lung's lower lobe on chest CT, consistent with either metastasis or a second primary (Figures 1A, 1B). Wedge resection of the lung tumor was performed.

**Figure 2:**
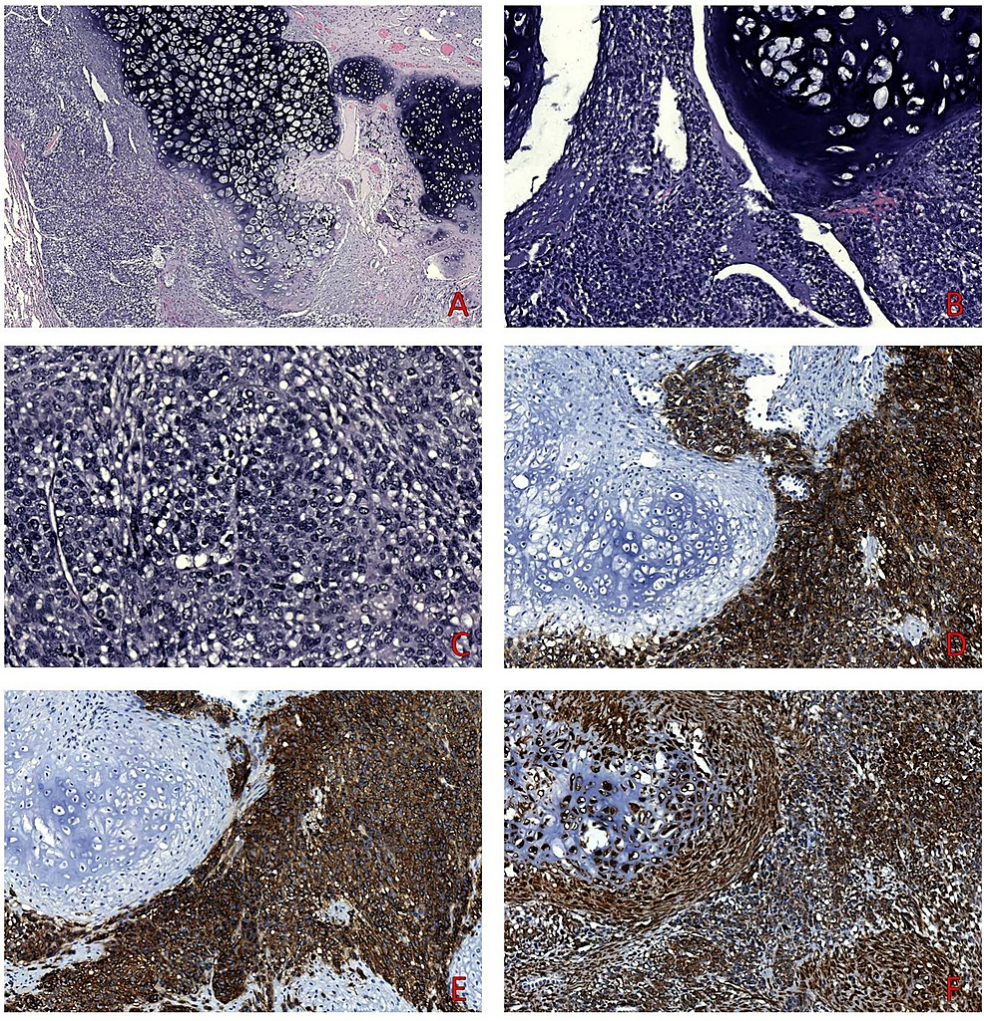
The lung metastasis showed a biphasic appearance consisting of epithelioid and chondrosarcomatous components (A-C). Epithelioid areas stained diffusely for DOG1 (D) and CD117 (E), while Vimentin showed uniform staining in all tumors cells (F).

The resection specimen was fixed in 10% buffered formalin for 24 hours. On gross examination, a well-circumscribed, solid, gray-white, 1.3-cm-large tumor was found; it was embedded in toto. Both hematoxylin and eosin (H&E) stained and immunostained sections from the previously operated gastric tumor were retrieved from the files of the Department of Pathology and compared to those of the lung tumor. This consisted of two components: an epithelioid one, with a solid architecture, high-grade atypia, and numerous mitotic figures, with an abrupt transition to an atypical chondrogenic component, consistent with chondrosarcoma (Figures 2A-2C). Thus, whereas the primary gastric tumor was a spindle-cell GIST, the recurrent tumor had both an epithelioid and a chondrosarcomatous component. The epithelioid component expressed DOG-1 (Figure 2D) and CD117 (Figure 2E), whereas both components were immunostained for Vimentin (Figure 2F); CKAE1/AE3, CK5/6, p63, TTF1, Napsin A, and CD99 immunostains were uniformly negative. Analysis through immunostains performed at the initial diagnosis showed both the primary gastric tumor and the recurrence to be positive for CD117, CD34, and Vimentin and negative for CKAE1/AE3 SMA, Desmin, and S100 protein. A diagnosis of a malignant pulmonary neoplasm with morphological and immunohistochemical features consistent with metastasis of the previously diagnosed gastric GIST, with heterologous chondrosarcomatous dedifferentiation, was rendered.

Mutational analysis with real-time PCR technique was performed on samples from the primary tumor, the local recurrence, and both differentiated and dedifferentiated areas of the pulmonary tumor, revealing no KIT or PDGFRA mutation. IM treatment was resumed. Four months later, abdominal CT showed a decrease in the size of the juxtapancreatic tumor. The patient is currently still on IM.

## Discussion

GISTs are the commonest mesenchymal tumors of the digestive tract, usually displaying spindle cell morphology, with epithelioid or mixed patterns being less frequent. All subtypes have uniform nuclei and usually display low mitotic activity [1].

Immunostaining for CD117, DOG1, and CD34 clinches the diagnosis of GIST; CD117 stains ca 95% of tumors, DOG1 75%-100%, and CD34 60%-70% of tumors [1]. Neoplastic cells may less often be decorated for SMA, S-100 protein, desmin, or keratin [17].

Most GISTs have KIT gene mutations, most commonly located in exon 11 and less often in exons 9, 13, or 17 [18-20]. PDGFRA gene mutations occur less frequently (10%-15% of GISTs) involving exons 12, 14, or 18 [21]. KRAS mutations have been reported in 5% of GISTs and may be related to a possible novel mechanism of primary resistance to IM [22]. Wild type KIT/PDGFRA/RAS GISTs may harbor succinate dehydrogenase deficiencies [23] or BRAF exon 15 V600E mutations [24,25].

Few cases of GIST dedifferentiation have previously been reported: a review of the pertinent English literature yielded 12 papers describing 26 cases with unusual morphological and/or immunohistochemical findings, either de novo or after therapy with TKIs [5-16]. Four papers described eight, five, three, and two cases, respectively, whereas the remaining eight were single case reports. Patient ages ranged from 23 to 75 years (median 55 years); 20 patients were male and six female. The primary tumor was located in the stomach (15 cases), small bowel (seven cases), colon (two cases), and rectum (one case), with a single retroperitoneal E-GIST. Demographic and clinicopathological data of all cases are summarized in Table 1.

**Table 1:** Demographic and clinical data of dedifferentiated GISTs M: male, F: female, LNs: lymph nodes, LR: local recurrence, NA: not available, NM: not mentioned, DOD: died of disease, AWD: alive with disease, ANED: Alive with no evidence of disease, mo: months, * tumors with de novo dedifferentiation, **previous IM treatment for chronic myeloid leukemia.

Case	Author	Year	Age	Sex	Tumor size	Tumor location	Metastases at presentation	LR/ Metachronous Metastases	Outcome (mo)
1	Pauwels et al. [5]	2005	37	M	NA	Stomach	No	Abdominal, disseminated	DOD
2	Pauwels et al. [5]	2005	46	M	17 cm	Small bowel	No	Liver	ANED 31
3	Pauwels et al. [5]	2005	73	M	7.5 cm	Rectum	No	Liver, peritoneum	AWD 88
4	Liegl et al. [9]	2009	53	M	15 cm	Small bowel	No	Liver, abdominal	DOD 87
5	Liegl et al. [9]	2009	39	F	6 cm	Small bowel	Liver, pancreas	Peritoneal, retroperitoneal, right ovary, left lung, para-aortic LNs, L1 vertebral body	DOD 22
6	Liegl et al. [9]	2009	35	F	10 cm	Stomach	No	Abdominal, pelvic	AWD 65
7	Liegl et al. [9]	2009	57	M	16 cm	Stomach	No	Abdominal	AWD 33
8	Liegl et al. [9]	2009	66	M	20 cm	Stomach	No	Peritoneal	ANED 33
9	Vassos et al. [10]	2011	62	F	20 cm	Stomach	No	No	ANED 28
10	Martz et al. [11]	2013	65	M	13 cm	Small bowel**	Retroperitoneal LNs	Liver, omental, LNs	NM
11	Antonescu et al. [8]	2013	23	M	NA	Stomach	Liver	Liver, peritoneal	NM
12	Antonescu et al. [8]	2013	40	F	8 cm	Stomach*	Peritoneum	Peritoneal	NM
13	Antonescu et al. [8]	2013	55	M	18 cm	Stomach*	No	No	NM
14	Antonescu et al. [8]	2013	48	M	5.5 cm	Stomach*	Peritoneum	Liver, peritoneal	NM
15	Antonescu et al. [8]	2013	58	M	6 cm	Rectum*	No	No	NM
16	Antonescu et al. [8]	2013	53	M	7 cm	Stomach*	Locoregional LNs	Peritoneal	NM
17	Antonescu et al. [8]	2013	60	M	7.5 cm	Small bowel	No	Liver, peritoneal	NM
18	Antonescu et al. [8]	2013	65	M	25 cm	Colon	No	Peritoneal	NM
19	Jung et al. [12]	2013	51	M	11 cm	Stomach*	Locoregional LNs, liver	No	ANED 10
20	Choi et al. [13]	2014	52	F	30 cm	Small bowel*	No	No	NM
21	Jiang et al. [6]	2015	47	M	14 cm	Stomach	Pelvis	Pelvic	AWD 48
22	Zhu et al. [7]	2015	57	M	12 cm	Retroperitoneum	No	LR after 15 mo	DOD 42
23	Jung et al. [14]	2017	72	M	7 cm	Stomach	Liver	Liver	AWD 72
24	Jung et al. [14]	2017	67	F	10 cm	Stomach	No	No	ANED 58
25	Li et al. [15]	2019	75	M	9.3 cm	Stomach	No	No	ANED 20
26	Shah et al. [16]	2021	64	M	8cm	Small bowel *	No	NM	NM
27	Present case	2021	52	M	10 cm	Stomach	Locoregional LNs	LR after 60 mo, lung	AWD 190

Pretreatment specimens were available in 18 cases: 16 had spindle-cell and the remaining two epithelioid morphology. The post-treatment aspect showed a well-differentiated spindle cell proliferation consistent with GIST next to a dedifferentiated high-grade malignancy, most frequently rhabdomyosarcomatous (eight cases) [6,9,15,16], with six cases showing epithelioid/pleomorphic differentiation [5,10,13], five cases nondescript anaplastic features [8] and one each showing epithelioid/tubulopapillary [5], undifferentiated pleomorphic sarcoma [12], and angiosarcomatous [8] features. The retroperitoneal E-GIST showed rhabdomyosarcomatous and chondrosarcomatous differentiation [7], whereas two cases showed changes in the immunophenotype without corresponding morphological alterations [14]. A case that deserves separate mention presented initially as a high-grade sarcoma of the small bowel, following long-term treatment with IM for chronic myeloid leukemia [11]. The diagnosis was confirmed by the molecular demonstration of a KIT exon 11 WK557-8 deletion [8].

In cases with available pretreatment samples, tumors showed almost always positive staining for CD117 and DOG1 and less often for CD34. In post-treatment tumors, the aforementioned markers were usually expressed in areas with GIST morphology and were lost or only focally retained in the dedifferentiated component. The expression of immunohistochemical markers in de novo dedifferentiated GISTs was similar to the post-treatment samples.

The case presented by Martz et al. showed strong diffuse staining for Vimentin, scattered positivity for CD34 and CAM5.2, MNF116, and multifocal positivity for EMA, CD117, DOG1, INI-1, ERG, and desmin. Lymphoid and melanoma markers were negative.

Regarding molecular findings, the three IM-resistant cases presented by Pauwels et al. had KIT exon 11 mutations [5]. In GISTs with rhabdomyoblastic differentiation, KIT exon 11 deletions [9], KIT exon 11 point mutation [6,9,15], and PDGFR exon 18 deletions [9] were detected in both components, without secondary mutations in the dedifferentiated component [9,14].

The molecular mechanism of tumor progression was investigated in three IM-treated and five IM-naïve tumors in the case series presented by Antonescu et al. [8]. Four of them had wild-type KIT, PDGFRA, and BRAF genes in both conventional and dedifferentiated components. Loss of a KIT gene copy due to haploinsufficiency was found in the dedifferentiated components of the three KIT-negative imatinib-resistant GISTs. KIT mutations in exons 11 were present in the two cases described by Jung et al. [14]. KIT mutations in exons 11 and 13 were present in two IM-treated tumors. Loss of heterozygosity or low-level KIT amplification was the most common finding in the dedifferentiated components [8]. KIT and KRAS mutations were found in the CD117, DOG1, and CD34 negative GIST with anaplastic dedifferentiation presented by Martz et al. [11].

Seven tumors with de novo dedifferentiation have been described [8,12,13]. Antonescu et al. found KIT mutations on exon 11 in three out of five cases. The other two tumors were wild-type with lower levels of KIT amplification as compared to that of the KIT-positive component with classic GIST morphology. KIT exon 11 mutation was detected in the case reported by Jung et al. [12]. GISTs of the small intestine reported by Choi et al. and Shah et al. were wild type [13,16].

The above findings suggest that dedifferentiation with loss of KIT expression may not be related to additional mutations in the original driver oncogene; dedifferentiation could result from alternative escape mechanisms driven by KIT-independent signaling pathways [1].

Follow-up information was available in 15 cases, ranging from 10 to 87 months. Six patients were alive with no evidence of disease, five were alive with disease, and four died of the disease.

GIST dedifferentiation might cause diagnostic problems for the inexperienced pathologist. Malignant "Triton" tumor, dedifferentiated liposarcoma, desmoid fibromatosis, or collision tumors are significant diagnostic problems. In difficult or doubtful cases, extensive sampling, detailed morphological analysis, appropriate use of immunohistochemical markers, and mutational analysis are the key to the correct diagnosis.

In our case, due to the biphasic (epithelioid and chondrosarcomatous) morphology, the differential diagnosis included sarcomatoid carcinoma of the lung; immunopositivity of neoplastic cells for CD117, DOG1, and negative staining for epithelial markers as well as for TTF1, Napsin A, and p63 resolved the diagnostic problem.

## Conclusions

We herein present the first (to our best knowledge) case of dedifferentiated GIST with heterologous, chondrosarcomatous differentiation, after treatment with IM. Awareness of the histological and immunohistochemical changes in GISTs, usually post-IM treatment, is essential to avoid severe diagnostic pitfalls. Furthermore, precise early diagnosis of dedifferentiation will lead to correct therapeutic decisions.
